# Trends in perinatal mortality and its risk factors in Japan: Analysis of vital registration data, 1979–2010

**DOI:** 10.1038/srep46681

**Published:** 2017-04-25

**Authors:** Maaya Kita Sugai, Stuart Gilmour, Erika Ota, Kenji Shibuya

**Affiliations:** 1 Department of Global Health Policy, Graduate School of Medicine, The University of Tokyo, Tokyo, Japan; 2 Global Health Nursing, Graduate School of Nursing Science, St. Luke’s International University, Tokyo, Japan

## Abstract

As Japan has achieved one of the lowest perinatal mortality rates (PMR), our study aims to estimate trends in and risk factors for perinatal mortality among singleton births in Japan. We used Japanese vital registration data to assess trends in and risk factors for perinatal outcomes between 1979 and 2010. Birth and death registration data were merged. An autoregressive integrated moving average model was fitted separately by sex to the PMR and the proportion of stillbirths. A multilevel Poisson regression model was used to estimate risk factors for perinatal mortality. Between 1979 and 2010 there were 40,833,957 pregnancies and 355,193 perinatal deaths, the PMR decreased from 18.86 per 1,000 all births to 3.25 per 1,000 all births, and the proportion of stillbirths increased from 83.6% to 92.1%. Key risk factors for perinatal mortality were low or high birth weight, prematurity and post maturity, and being from poorer or unemployed families. A higher proportion of excess perinatal deaths could be averted by effective policies to prevent stillbirths and improved research into their interventions and risk factors. As the cost and challenge of maintaining perinatal mortality gains increases, policies need to be targeted towards higher risk groups and social determinants of health.

The perinatal mortality rate (PMR) is an important indicator of the quality of obstetric care during pregnancy^1^,^2^. Perinatal mortality includes deaths that occur due to obstetric events, stillbirths and early neonatal mortality (ENM) occurring within seven days after birth^3^. Neonates are at most risk of dying in their first week of birth, and globally three-quarters of neonatal deaths occur in the first week^4^. Even in high-income settings where the quality of medical services and resources is high, policies to reduce the PMR are still needed^5^,^6^, and as much as 25% of perinatal mortality in developed countries is estimated to be preventable by achievement of optimal standards of care quality^1^. Japan has achieved the lowest PMR amongst developed nations^7^, contributing to the remarkable improvement in Japan’s overall health status in the 20^th^ century^8^. This achievement required large reductions in the PMR compared to other countries^9^, through declines in both the rate of stillbirths and the early neonatal mortality rate (ENMR)^10^,^11^, which was 0.9 (0.7 to 1.2) deaths per 1,000 live births in 2013^12^. This reduction was achieved through improvements in medical care^9^ and public health interventions focusing on maternal and child health, including the introduction of new drugs, vaccines, diagnostics, and procedures^12^. These interventions increased the survival rate of preterm and extremely low birth weight neonates, resulting in health gains that could not have been achieved before^9^.

Perinatal death can occur due to fetal, neonatal and/or maternal causes, making disease risks interrelated and complex^13^. Lessons learnt in Japan will be valuable for other nations: An up-to-date estimate of the trend in perinatal mortality will provide a clearer picture of how Japan has reduced mortality over the decades, and understanding of its risk factors can help to aid in future clinical and public health policy development. A comprehensive analysis of perinatal mortality in singleton births over time will also enable a consistent and up-to-date comparison with trends and risk factors in other countries. This study assessed the trend in perinatal mortality from 1979 to 2010 using a comprehensive set of individual birth and death records from the Japanese vital registration system, and examined risk factors for perinatal mortality using a multi-level logistic regression model.

## Methods

### Data source

Data on all live births, early neonatal deaths, and stillbirths occurring between 1979 and 2010 were provided by the Ministry of Health, Labour and Welfare (MHLW) in Japan. These data included information such as birth weight, maternal age, gestational age and parity at the municipality level^14^,^15^.

Only singletons and births with missing multiplicity data were assessed in this study, as multiple births are known to have different risk factors for PMR compared to single births^16^. Observations with missing prefecture data, gestational age or sex were excluded. In the analysis, observations were dropped when maternal age was missing, or when there were more than eight previous births or deaths.

### Availability of data and materials

The data used in this research was obtained from the Japan vital registration database maintained by the MHLW. The conditions of access to this data preclude sharing of the data with researchers not directly involved in this project.

### Outcome and covariates

Perinatal mortality was defined as stillbirths occurring at or after 22 weeks of gestational age or any early neonatal death occurring within 7 days after birth^3^,^17^. The PMR was calculated as the total of all stillbirths and early neonatal deaths, divided by the sum of stillbirths and live births (hereafter referred to as “all births”).

For the risk factor analysis sex, gestational age, maternal age, birth weight, parity, previous stillbirth history, household occupation, and year were included as covariates. Gestational age was measured as the time between the last menstrual period and the date of delivery. Gestational age was divided into four categories: early preterm (<34 weeks), late preterm (34 to 36 weeks), term (37 to 41 weeks), and post mature (>41 weeks). Maternal age was divided into six categories: 15–19, 20–24, 25–29, 30–34, 35–39, and over 40. Birth weight was recorded per 100 grams during 1979–1991, and in grams after 1992. High birth weight was defined to be a birth weight over 4,000 grams, and stillbirths and neonates between 2,500 grams and 4,000 grams were defined to be normal birth weight. We categorized low birth weight into three categories: under 1,000–grams, 1,000–1,499–grams, and 1,500–2,500 grams^18^. Previous experience of stillbirth was defined as the number of past stillbirths after 20 weeks’ gestational age before 1995, or after 22 weeks for post-1995 data. Occupational status was categorized into six groups according to the occupation of the parent with higher income: full-time or part-time farmer; self-employed; full-time employee of a company or private concern with less than 100 employees; other full-time employee; other or unemployed (e.g. daily or dispatched workers, students); and unknown. History of birth and stillbirth were dichotomized.

### Merging process

Because an individual identifier was not available in the datasets, the birth and death datasets could not be merged on identifying data, and were merged on sets of variables. The datasets were merged separately for 1975–1994 and 1995–2010, due to differences in available data. Only data from 1979–2010 was merged, to provide consistent gestational age measurements for all years. Details of the merging process are provided in the appendix. As some individuals, hereafter defined as *duplicate births*, could not be uniquely identified using only the available sets of variables, especially in larger municipalities, we merged deaths to births that could be uniquely identified (hereafter defined as *unique births*) first, then merged the duplicate births to deaths. Deaths were assigned to duplicate births based on mortality probabilities calculated from a multi-level Poisson regression of early neonatal mortality run on the data set of unique deaths and births. This model included sex, maternal age, gestational age, birth history, and stillbirth history. The duplicated birth with the highest probability of early neonatal death was merged with the death observation. Finally, the stillbirth data was appended to create a full dataset of births, deaths, and stillbirths.

### Statistical analysis

A time-series analysis was conducted to assess the annual trends in PMR and the proportion of perinatal deaths that were stillbirths, with the natural logarithm of perinatal mortality rate and the inverse logistic transformation of the proportion of stillbirths as outcomes. To obtain stationarity, the outcomes in both analyses were differenced. The differenced natural logarithm of PMR is equivalent to the ratio of PMR between two consecutive time points, while the differenced inverse logistic transformation is equivalent to the odds ratio between two consecutive time points. The annual percentage change of the PMR and the proportion of stillbirths were calculated from the rate ratio of the PMR and the odds ratio of the proportion of stillbirths. An autoregressive integrated moving average (ARIMA) model was fitted separately for male and female births, because sex differences have previously been observed in the PMR^19^. An autoregressive function of lag 1 (AR (1)) was used to fit both models. The autocorrelation function and the partial autocorrelation function were estimated for both analyses, and the residuals from the models were tested to confirm no significant auto-correlation within the first 13 lags.

A multi-level Poisson regression was conducted using the generalized linear latent and mixed model (GLLAMM)^20^,^21^, with a random effect for prefecture and 24 integration points. Both the time-series analysis and the multilevel analysis were adjusted using a step function to account for the downward step in mortality due to the change in definition of the abortion law in 1991 from 24 weeks of gestational age to 22 weeks of gestational age. The step function was used to account for bias due to the implementation of the change in abortion law over time. This step function was entered into the model as a variable that is 0 before the change in law, and 1 afterwards, and enables the model to incorporate a uniform increase or decrease in mortality due to the redefinition of abortion under the law. The use of a step function to model uniform changes in time series models is routine practice in time series analysis^22^. Its coefficient can be interpreted as a constant multiplier of the mortality rate (or a constant multiplier on the odds of stillbirth), after the change in law compared to before. A value below 1 indicates that the law led to an overall reduction in the rate of mortality (or the odds of stillbirth) while a number greater than one indicates an overall increase in the rate of mortality (or the odds of stillbirth) after the law was changed. This step function is hereafter referred to as the “abortion law threshold step.” For supplementary analysis, time-series analysis and multilevel analysis were additionally conducted for perinatal mortality after 1992, to check how the results may differ when the analysis is restricted to just the period after the abortion law was changed. All analysis and data preparation were conducted in Stata/MP 12.

## Results

**Figure 1:**
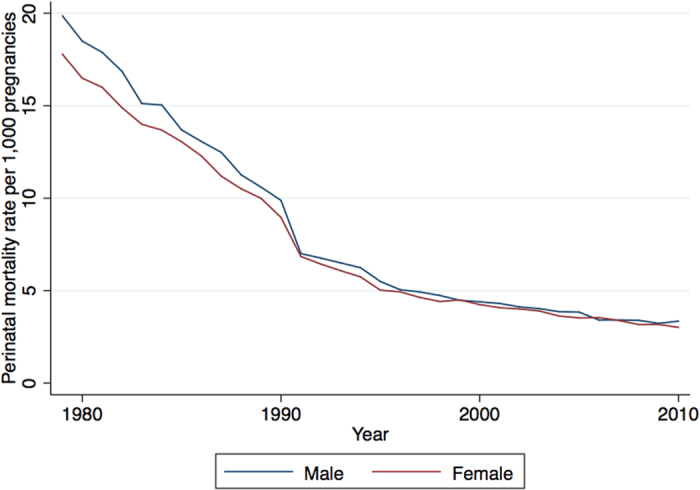
Annual trend in perinatal mortality rate per 1000 all pregnancies by sex, 1979–2010.

From January 1979 to December 2010, there were 40,833,957 pregnancies and 355,193 perinatal deaths, giving a PMR ranging from 19.86 (95% CI: 19.57–20.16) per 1,000 all births in 1979 to 3.35 (95% CI: 3.20–3.50) per 1,000 all births in 2010 for male, and 17.79 (95% CI: 17.50–18.08) per 1,000 all births to 3.01 (95% CI: 2.86–3.16) per 1,000 all births for female over the same period. Figure 1 shows the decline in PMR for both male and female births.

The merging rate reached 92.7% for all years (Supplementary Figures 1 and 2). We excluded 50,693 congenitally malformed babies, which comprised 13.0% of all deaths.

Table 1 shows the results of the annual time-series analysis of perinatal mortality, stratified by sex. There was an annual reduction in the PMR of 4.7% (95% CI: 3.6–5.8%) for males, and 4.9% (95% CI: 3.9–5.8%) for females.

**Figure 2:**
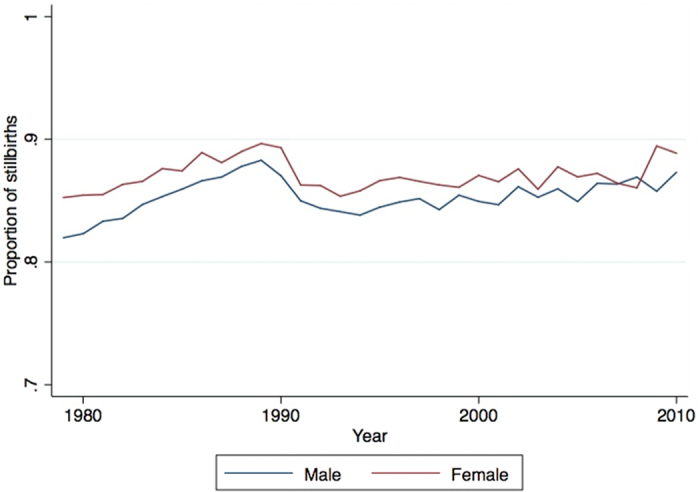
Annual trend in proportion of stillbirths by sex, 1979–2010.

Figure 2 shows the trend in the proportion of deaths due to stillbirth between 1979 and 2010, and Table 2 shows the results of annual time-series analysis of the odds of stillbirth over this period. There was an overall increase in the proportion of stillbirths from 83.6% to 92.1%, giving an annual increase in the odds ratio of stillbirth of 1.02 (95% CI: 1.01–1.04) for male and 1.02 (95% CI: 1.00–1.04) for female fetuses. For both time-series analysis of the PMR and the odds ratio of stillbirth, the effect of the step due to change in the stillbirth criteria was not statistically significant. Time-series analysis of the PMR and the odds of stillbirth was also conducted restricting the data to 1992–2010, the years after the change in abortion law in 1991, and is presented in the appendix as Supplementary Tables 1 and 2. Supplementary Table 1, showing results of annual time-series analysis of the PMR after 1992, was numerically similar to the results in Table 1. However the annual change in the odds of stillbirth, was statistically non-significant over this shorter period, even though the results were numerically similar, likely due to the shorter time series used for this supplementary analysis.

Table 3 shows the results of the multi-level Poisson regression. The risk ratio for high birth weight was 5.88 (95% CI: 5.74–6.04) compared to normal birth weight neonates. Low birth weight neonates had a risk ratio for perinatal mortality of 5.97 (95% CI: 5.88–6.07), and this risk ratio increased to 40.44 (95% CI: 39.66–41.23) for extremely low birth weight neonates. Risk of perinatal mortality was increased in earlier and later gestational ages, ranging from 10.22 (95% CI: 10.03–10.40) for early premature to 4.57 (95% CI: 4.50–4.65) for late premature and 2.55 (95% CI: 2.48–2.63) for post mature compared to term neonates.

The mortality profile followed a J-curve shape over age categories, with maternal age 25–29 at the minimum of the curve. Disparities in perinatal mortality due to casual employment or unemployment were greater than the effects of maternal age, nulliparity, previous experience of stillbirth or infant sex, indicating a strong disparity in birth outcomes by occupational categories. The abortion threshold step contributed to an 8% reduction in the PMR. The multilevel analysis was conducted for years after the change in abortion law in 1991, as presented in Supplementary Table 3. Results from this analysis were numerically similar to the results in Table 3, justifying the use of the abortion threshold step in the model for the analysis of all years.

## Discussion

This is the first attempt to analyze systematically the trend in perinatal mortality in Japan between 1979 and 2010. Using a complete national registry dataset, our updated study identified higher values of the PMR than an earlier estimate of perinatal mortality among singletons of 11.0 per 1,000 live births in 1980 and 4.9 per 1,000 live births in 1991^19^. Our study found an annual decrease in perinatal mortality and an increasing proportion of stillbirths, suggesting a slower decline in stillbirth rates compared to ENM rates, as both ENM rates and stillbirth rates are decreasing^23^. The faster decline in ENM rates could be explained by increased capacity of NICUs improving effective interventions and quality of care to save lives of neonates in Japan. This shows an increased need to focus on decreasing stillbirth rates in Japan, a problem that has also been identified at a global level^24^. Stillbirths are a neglected issue, and not addressed in the Millennium Development Goals (MDG) 4 or in the global burden of disease (GBD) study^24^ before 2015, and focusing primarily on infant and early neonatal mortality will deliver decreasing gains in perinatal mortality for developed countries like Japan where the majority of perinatal mortality is due to stillbirth. Knowledge of the cause of stillbirths is limited compared to ENMs. For example, unavailability of high-quality data for cause of stillbirths, lack of early detection of stillbirth risk and interventions during pregnancy^25^, and unaddressed modifiable risk factors for stillbirth are key problems^26^. Despite this limited knowledge, some modifiable risk factors such as overweight, obesity, maternal age, and smoking have been identified and if tackled could contribute to a reduction in stillbirths^26^. A higher proportion of excess perinatal deaths could be averted by effective policies to prevent stillbirths and improved research into interventions targeting stillbirths and their major risk factors^26^,^27^,^28^,^29^.

While previous studies have simply catalogued perinatal mortality by different basic risk groups, we have conducted a multilevel analysis, applied a Poisson distribution instead of using an ordinary-least-squares method, and used a complete set of Japanese vital statistics. Previous, unadjusted analyses found sex differences in perinatal mortality among singleton births between 1980–1991^19^. We have found that these sex differences in Japanese singletons are now very small, representing just 0.25 perinatal deaths per 1,000 in 2010, or approximately 300 perinatal deaths per year. Instead of infant sex, key risk factors identified in this study were birth weight, gestational age, and household occupation. There is an increasing proportion of low birth weight (LBW) neonates in Japan^30^,^31^, and a reduction in mean birth weight of more than 150 g from 1980 to 2003^31^. From 1980 to 2009 there has been a slight increase in the proportion of preterm live births from 3.8% to 4.7%, whereas there has been a decrease in numbers of post mature neonates from 4.5% to 0.4% of all live births and a corresponding increase in the proportion of term live births from 91.7% to 94.9%^32^. This increase in the proportion of preterm births, combined with trends in birth weight, indicates both a shift in the balance of risks and in the costs associated with preventing early neonatal mortality.

Promoting strategies known to be effective to prevent preterm birth, such as caesarian delivery and labour induction^33^, and better management of preterm babies, are essential if Japan is to maintain its momentum in reducing perinatal mortality. Reversal of this LBW trend is also essential to reduce the long-term negative health and development outcomes of LBW, such as cardiovascular disease and diabetes^34^,^35^,^36^,^37^,^38^,^39^,^40^,^41^. Managing high-risk pregnancies will become a greater challenge, as the numbers of older mothers, preterm births, and LBW births increase. Especially, given that maternal age is likely to continue to increase along with the aging of Japanese society, delayed marriages^42^ and decreasing fertility rates^43^, management of risk factors such as gestational hypertension, low birth weight and preterm birth will need to be improved.

Occupational status was also associated with perinatal mortality. Occupational status is a partial proxy for socioeconomic status in Japan and there is evidence that it is associated with inequity in adult mortality^44^, so the finding that it is also associated with perinatal mortality is a matter of concern. Research in other high income countries^45^,^46^,^47^ has also found persistent inequality in health outcomes across socioeconomic groups^48^,^49^,^50^ and within ethnic minorities^51^,^52^, even where inequality in access is not observed. Interventions aimed at improving access to antenatal care have not been found to reduce this inequality^45^, which is likely due to social determinants of health rather than health system factors^53^. Similarly to other countries such as the UK^54^, Japan will need to address risk factors such as congenital anomaly^55^, LBW^51^,^56^, preterm birth^51^,^57^, and small-for-gestational-age^51^, which are known to be associated with area-level social deprivation. Socioeconomic inequality may also affect birth outcomes through increased prevalence of smoking^58^, nutritional deficiencies such as obesity, and overcrowding and other poor living conditions associated with sudden infant death syndrome (SIDS)^59^.

Finally, we found an increasing proportion of perinatal mortality was due to stillbirth. This emphasizes the need to focus on decreasing stillbirth rates in Japan, a problem that has also been identified at a global level^24^. Stillbirths are a neglected issue^24^, and a focus on infant and early neonatal mortality will deliver decreasing gains in perinatal mortality for developed countries like Japan where the majority of perinatal mortality is due to stillbirth. Improved research into interventions targeting stillbirths and their major risk factors^26^,^27^,^28^,^29^ is necessary^60^ in order to develop effective policies to prevent stillbirths. Increasing screening and treatment for syphilis^61^, improving access to planned caesarean section for breech delivery^61^ putting more focus on comprehensive emergency obstetric care^60^,^61^ and induction of labor for post-term pregnancy^60^, are interventions with evidence of contribution to stillbirth reduction. Other developed nations following Japan’s trajectory in perinatal mortality need to address modifiable risk factors for reduction of stillbirths such as overweight, obesity, maternal age, and smoking. There is an absence of access to high-quality data to identify the cause of stillbirths, and lack of early detection of stillbirth risk and interventions during pregnancies^25^, and these gaps in knowledge need to be addressed in Japan in order to drive reductions in the PMR in future.

Our findings have several limitations. Personal risk factors such as maternal gestational weight gain, pre-existing conditions, smoking status and alcohol intake were missing, as they were not recorded in vital statistics throughout all years. It was not possible to incorporate a history of past early neonatal deaths, as this data is not recorded in the national vital registration system. As stillbirths constitute more than 80% of all perinatal deaths, this variable is likely to be broadly representative of past history of perinatal mortality in both regression analyses, but its incompleteness means it should be viewed with caution. Second, the mode of delivery was also unknown for all observations, so we cannot assess the impact of caesarian section, which may confound the effect of gestational age on perinatal mortality. The proportion of caesarian deliveries has increased from 17.7% in 2007 to 18.6% in 2011^62^. However, no significant relationship has been found between caesarian section and PMR in Japan^62^, so this limitation may not have a significant effect on the risk factor model presented here. We could not fully account for the effect of different coding systems and measurement style between each health facility, or directly adjust for local policies, although these unobserved effects were likely partially adjusted for through the use of a prefecture-level random effect. Coding systems also changed over time. The gestational age definition for recording past history of stillbirth changed in 1995, giving this risk factor differing salience in the two periods. The Ministry does not yet have a sufficiently standardized reporting system, and further efforts need to be made to standardize the reporting of deaths and to record other, essential risk factors such as maternal weight and past history of neonatal or infant death. Finally, we could not conduct sensitivity analysis of the effect of merging on the regression model, since to run even a small bootstrapping process on the model would take more than a year. This means that any biases introduced by matching non-unique births to deaths on ENM mortality profiles cannot be assessed definitively. However, only about 30% of early neonatal deaths were merged in this way, and it is our judgment that this small number was unlikely to have seriously biased the findings of our regression model.

## Conclusion

We found that PMR decreased, and proportion of stillbirths increased over time in Japan from 1979 to 2010. This constant decline in PMR translates to gradually smaller gains in absolute numbers of lives saved, and interventions will need to become more complex to continue to improve the PMR. In order to further reduce the PMR, targeting of risk factors will be needed along with an increased focus on interventions targeting stillbirths. With only 400 preventable early neonatal deaths per year, further gains in perinatal mortality will be costly and challenging, but through increasing attention to specific risk factors and stillbirths Japan can maintain its historically high rate of improvement of perinatal mortality.

## Additional Information

**How to cite this article:** Sugai, M.K. *et al*. Trends in perinatal mortality and its risk factors in Japan: Analysis of vital registration data, 1979–2010. *Sci. Rep.* **7**, 46681; doi: 10.1038/srep46681 (2017).

**Publisher's note:** Springer Nature remains neutral with regard to jurisdictional claims in published maps and institutional affiliations.

## Supplementary Material

Supplementary Information

## Figures and Tables

**Table 1:** ARIMA time-series analysis of perinatal mortality rate by sex, 1979–2010.

	Value	95% CI	P value
Male			
Rate ratio (Annual)	0.953	0.942–0.964	<0.001
Abortion threshold step	1.346	0.523–3.460	0.5
AR (1)	−0.149	−0.522–0.224	0.4
Female			
Rate ratio (Annual)	0.951	0.942–0.961	<0.001
Abortion threshold step	1.252	0.354–4.429	0.7
AR (1)	−0.096	−0.738–0.545	0.8

AR (1): Autoregressive function of lag 1.

CI: Confidence interval.

**Table 2:** ARIMA time-series analysis of proportion of stillbirths out of all perinatal deaths by sex, 1979–2010.

	Value	95% CI	P value
Male			
Odds ratio (Annual)	1.021	1.005–1.038	<0.01
Abortion threshold step	1.321	0.896–1.949	0.2
AR (1)	−0.556	−0.962–−0.149	<0.01
Female			
Odds ratio (Annual)	1.023	1.002–1.044	<0.05
Abortion threshold step	1.406	0.225–8.796	0.7
AR (1)	−0.499	−0.892–−0.106	<0.05

AR (1): Autoregressive function of lag 1.

CI: Confidence interval.

**Table 3:** Multilevel regression model of risk factors for perinatal mortality.

	Risk ratio	95% CI	P value
Sex			
Male	1.00		N/A
Female	0.95	0.95–0.96	<0.01
Birth weight			
Normal (2,500–4,000 g)	1.00		N/A
High (>4,000 g)	5.88	5.74–6.04	<0.01
Low (2,000–2,499 g)	5.97	5.88–6.07	<0.01
Very low (1,500–1,999 g)	17.81	17.45–18.18	<0.01
Extremely low (<1,500 g)	40.44	39.66–41.23	<0.01
Maternal age			
25–29	1.00		N/A
15–19	1.10	1.09–1.12	<0.01
20–24	1.03	1.02–1.04	<0.01
30–34	1.03	1.03–1.04	<0.01
35–39	1.12	1.11–1.13	<0.01
Over 40	1.29	1.27–1.32	<0.01
Gestational age			
Term (37–41 weeks)	1.00		N/A
Early preterm (<34 weeks)	10.22	10.03–10.40	<0.01
Late preterm (34–36 weeks)	4.57	4.50–4.65	<0.01
Post mature (>41 weeks)	2.55	2.48–2.63	<0.01
Experience of past births			
Nulliparous	1.00		N/A
Primiparous	1.07	1.06–1.08	<0.01
Experience of past stillbirth			
No	1.00		N/A
Yes	1.10	1.08–1.11	<0.01
Household occupation			
Large company	1.00		N/A
Farmer	1.07	1.06–1.08	<0.01
Self-employed	1.04	1.03–1.05	<0.01
Small company	1.00	0.99–1.01	0.5
Casual/other	1.23	1.22–1.24	<0.01
Unemployed or unknown	1.44	1.40–1.49	<0.01
Year	0.96	0.96–0.96	<0.01
Abortion threshold step	0.92	0.91–0.94	<0.01

CI: Confidence interval.
