# Supplementary material for: Trends in perinatal mortality and its risk factors in Japan: Analysis of vital registration data, 1979–2010
Source: Sci Rep. 2017 Apr 25;7:46681. doi: 10.1038/srep46681 (PMC5404230; doi:10.1038/srep46681)
Supplement: Supplementary Information [file srep46681-s1.pdf]

Supplementary Information

Trends in perinatal mortality and its risk factors in Japan:

Analysis of vital registration data, 1979-2010

Maaya Kita[1], Stuart Gilmour[1], Erika Ota[2], Kenji Shibuya[1]

1. Department of Global Health Policy, Graduate School of Medicine, The University of Tokyo, Japan
2. Global Health Nursing, Graduate School of Nursing Science, St. Luke's International University, Tokyo, Japan

Supplementary Figure 1. Data preparation flowchart for 1979-1994

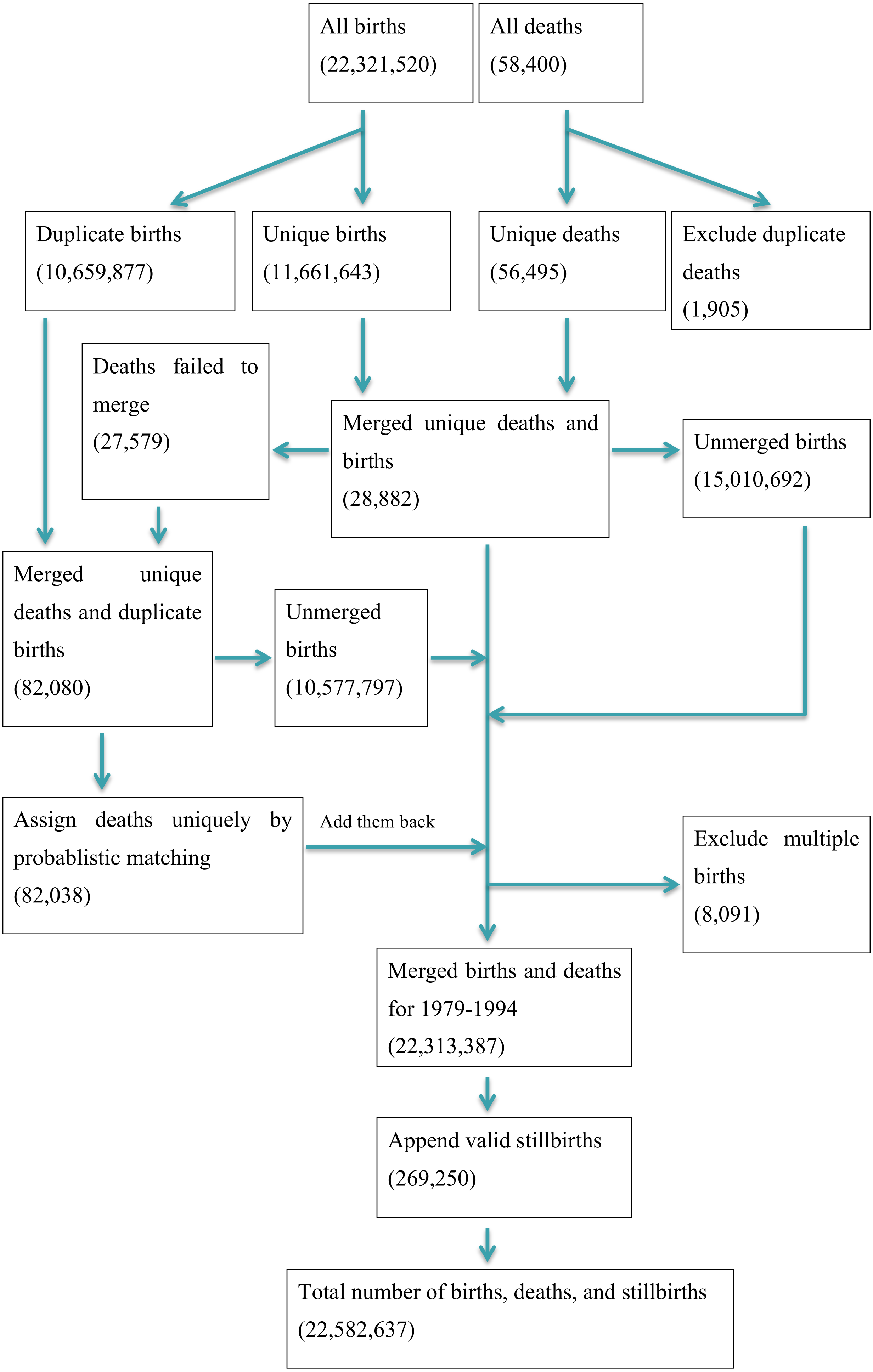

Supplementary Figure 2. Data preparation flowchart for 1995-2010

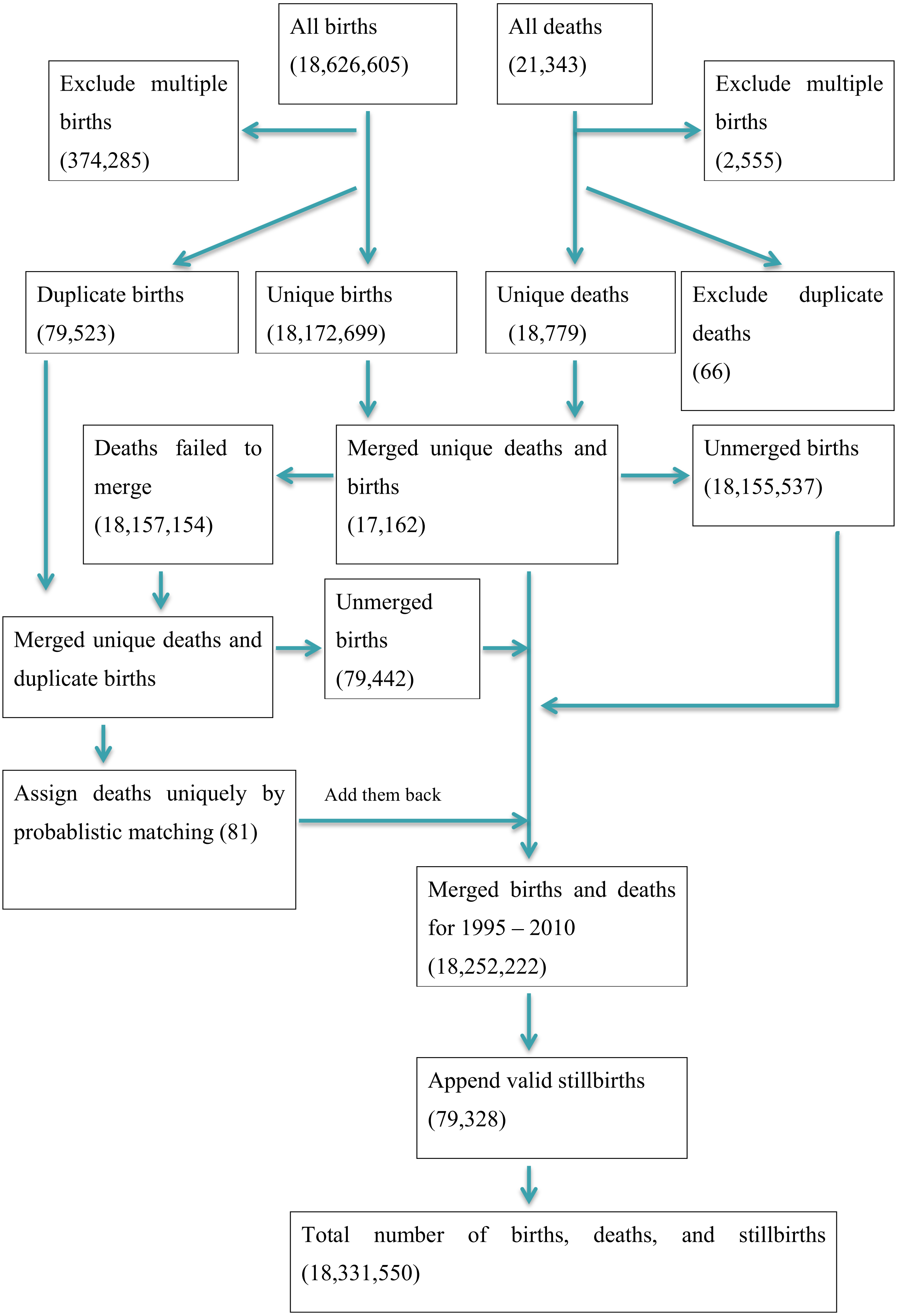

Supplementary Table 1. ARIMA time-series analysis of perinatal mortality rate by sex, 1992-2010

	Value	95% CI	P value
Male			
Rate ratio (Annual)	0.961	0.944 – 0.979	<0.001
AR (1)	-0.103	-0.584 – 0.378	0.7
Female			
Rate ratio (Annual)	0.956	0.944 – 0.974	<0.001
AR (1)	-0.178	-1.438 – 1.084	0.8

AR (1): Autoregressive function of lag 1

CI: Confidence interval

Supplementary Table 2. ARIMA time-series analysis of proportion of stillbirths out of all perinatal deaths by sex, 1992-2010

	Value	95% CI	P value
Male			
Odds ratio (Annual)	1.011	0.998 – 1.025	0.10
AR (1)	-0.745	-1.135 – -0.354	<0.01
Female			
Odds ratio (Annual)	1.017	0.978 – 1.057	0.40
AR (1)	-0.490	-1.063 – 0.084	0.09

AR (1): Autoregressive function of lag 1

CI: Confidence interval

Supplementary Table 3. Multilevel regression model of risk factors for perinatal mortality, 1992-2010

	Risk ratio	95% CI	P value
Sex			
Male	1.00		N/A
Female	0.97	0.96 – 0.99	<0.01
Birth weight			
Normal (2,500 – 4,000g)	1.00		N/A
High (>4,000g)	2.43	2.25 – 2.63	<0.01
Low (2,000 – 2,499g)	4.90	4.77 – 5.03	<0.01
Very low (1,500 – 1,999g)	12.88	12.39 – 13.38	<0.01
Extremely low (<1,500g)	42.96	41.44 – 44.53	<0.01
Maternal age			
25-29	1.00		N/A
15-19	1.11	1.07 – 1.15	<0.01
20-24	1.01	0.99 – 1.03	0.38
30-34	1.01	0.99 – 1.02	0.32
35-39	1.04	1.02 – 1.07	<0.01
Over 40	1.18	1.14 – 1.22	<0.01
Gestational age			
Term (37 – 41 weeks)	1.00		N/A
Early preterm (<34 weeks)	12.64	12.23 – 13.07	<0.01
Late preterm (34 – 36 weeks)	5.36	5.21 – 5.51	<0.01
Post mature (>41 weeks)	3.27	3.04 – 3.52	<0.01
Experience of past births			
Nulliparous	1.00		N/A
Primiparous	1.04	1.03 – 1.05	<0.01
Experience of past stillbirth			
No	1.00		N/A
Yes	1.12	1.08 – 1.16	<0.01
Household occupation			
Large company	1.00		N/A

Farmer	1.05	1.01 – 1.08	<0.05
Self-employed	1.05	1.02 – 1.07	<0.01
Small company	0.94	0.92 – 0.95	<0.01
Casual/other	1.36	1.34 – 1.39	<0.01
Unemployed or unknown	1.42	1.37 – 1.47	<0.01
Year	0.96	0.96 – 0.96	<0.01

CI: Confidence interval
